# Tangutidines A–C, Three Amphoteric Diterpene Alkaloids from *Aconitum tanguticum*

**DOI:** 10.1007/s13659-021-00310-3

**Published:** 2021-05-12

**Authors:** Hao-Yi Li, Bing-Chao Yan, Li-Xin Wei, Han-Dong Sun, Pema-Tenzin Puno

**Affiliations:** 1 State Key Laboratory of Phytochemistry and Plant Resources in West China, Kunming Institute of Botany, Chinese Academy of Sciences, and Yunnan Key Laboratory of Natural Medicinal Chemistry, Kunming, 650201 People’s Republic of China; 2 University of Chinese Academy of Sciences, Beijing, 100049 People’s Republic of China; 3 Qinghai Provincial Key Laboratory of Tibetan Medicine Pharmacology and Safety Evaluation, Northwest Institute of Plateau Biology, Chinese Academy of Sciences, Xining, 810008 People’s Republic of China

**Keywords:** *Aconitum tanguticum*, Amphoteric diterpene alkaloids, Tangutidine, Cytotoxic activity

## Abstract

Three new diterpene alkaloids, tangutidines A–C (**1**–**3**), and four known alkaloids (**4**–**7**) were isolated from the whole plant of *Aconitum tanguticum*, from which amphoteric diterpene alkaloids (**1**–**3**) were obtained for the first time. The structures of **1**–**3** were elucidated by detailed interpretation of spectroscopic data, including MS and NMR data. All of them were evaluated for their cytotoxic activities.

**Supplementary Information:**

The online version contains supplementary material available at 10.1007/s13659-021-00310-3.

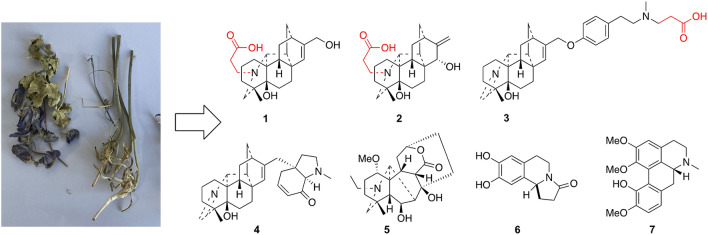

## Introduction

**Fig. 1:**
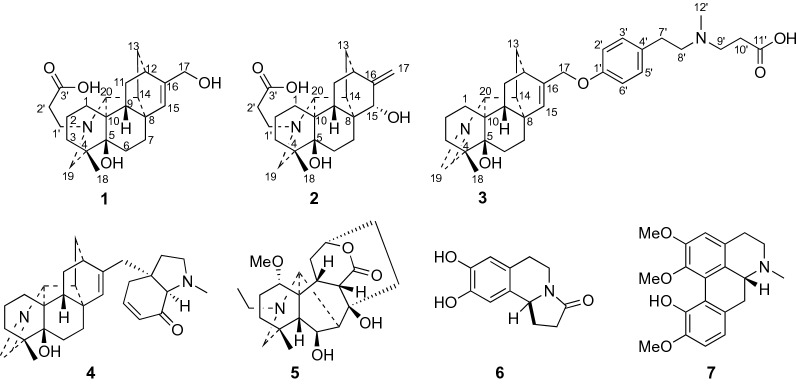
Structures of compounds **1**–**7**

Plants in the genus *Aconitum* of the family Ranuculaceae are abundant in C_19_- and C_20_-diterpene alkaloids with diverse structural scaffolds and important biological activities, which have long attracted scientists’ attention from chemistry and pharmacology communities [1–3]. *Aconitum tanguticum* (Maxim.) Stapf is mainly distributed in Tibet, Qinghai, Gansu, Sichuan and Yunnan Provinces in China [4]. Its whole plant has long been used as a traditional Tibetan medicine for treating fever caused by various infectious diseases, influenza and poisoning for thousands of years [5]. In a classic Tibetan Medical book *Sman dpyad zla ba`i rgyal po*, *A*. *tanguticum* is firstly recorded as one of the least toxic plants among other species in the genus *Aconitum* [5]. Previous phytochemical investigation of *A*. *tanguticum* showed that it contained diterpene alkaloids, flavonoids, phenolic acids, glycosides, etc. [6–16]. However, most of the studies on diterpene alkaloids in *A*. *tanguticum* focused on its fat-soluble part and few focused on the water-soluble part. In our phytochemical investigation of the whole plant of *A. tanguticum*, it resulted in the discovery of three new C_20_-diterpene alkaloids with carboxyl groups, tangutidines A–C (**1**–**3**) from the *n*-BuOH extract, together with four known alkaloids (**4**–**7**) (Fig. 1). In this paper, we reported their isolation, structure determination, and cytotoxicity.

## Results and Discussion

**Fig. 2:**
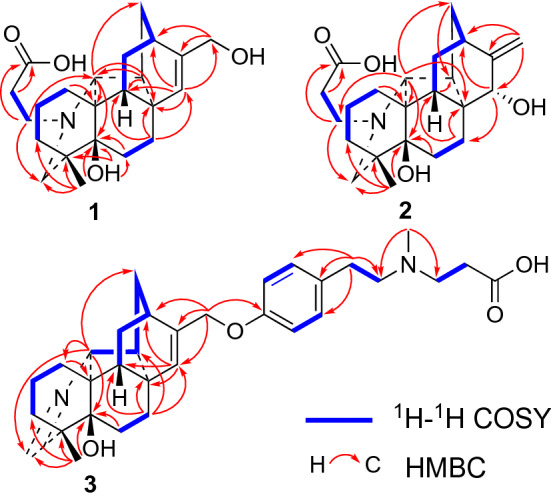
Key HMBC and ^1^H-^1^H COSY correlations of compounds **1**–**3**

**Fig. 3:**
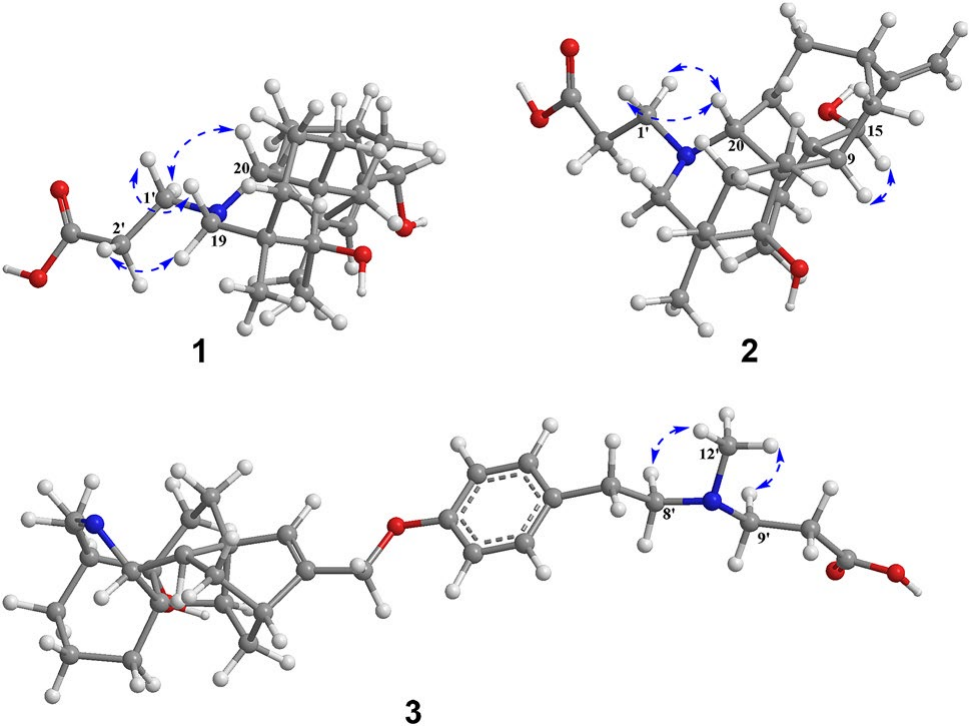
Key ROESY correlations of compounds **1**–**3**

Tangutidine A (**1**) was isolated as colorless oil with a molecular formula of C_23_H_33_NO_4_, which was determined by HRESIMS ([M+H]^+^ at *m/z* 388.2483, calcd 388.2482) with eight degrees of unsaturation. Its IR spectrum showed absorptions for hydroxyl (3428 cm^−1^) and carboxyl (1603 cm^−1^) groups. Analysis of the ^1^H, ^13^C NMR, DEPT and HSQC spectra of **1** revealed the existence of one olefinic bond (*δ*_H_ 5.83, br s; *δ*_C_ 128.6, d; 150.7, s), one oxygenated methylene group (*δ*_H_ 4.46, d, *J* = 2.2 Hz; *δ*_C_ 63.4, t), one tertiary methyl group (*δ*_H_ 1.15, s; *δ*_C_ 24.9, q), and four *sp*^3^ quaternary carbon (one oxygenated) (Table 1). All the mentioned evidence suggested that **1** was a hetidine-type diterpene alkaloid containing three extra carbons. Compared with the structure of naviculine A [8] bearing a double bond between C-19 and N-atom, **1** had an extra 3-*N*-propanoic acid moiety and two hydroxyl groups located at C-5 and C-17, respectively. The fragment C-1'/C-2'/C-3' was identified by the ^1^H–^1^H COSY correlation of H_2_-1'/H_2_-2', along with the HMBC correlation from H_2_-1'a (*δ*_H_ 3.31, m) to C-3' (*δ*_C_ 178.7, s). The connection between the fragment C-1'/C-2'/C-3' and N-atom was confirmed by the key HMBC correlation from H_2_-1'a (*δ*_H_ 3.31, m) to C-20 (*δ*_C_ 79.4, d) (Fig. 2) and the key ROESY correaltions from H_2_-1'a to H-20 (*δ*_H_ 2.70, s), H_2_-1'b (*δ*_H_ 2.99, m) to H-19b (*δ*_H_ 2.62, m) as well as H_2_-2' (*δ*_H_ 2.80, m) to H-19a (*δ*_H_ 3.11, br s) (Fig. 3). The key HMBC correlations of H-18 (*δ*_H_ 1.15, s) and H-6 (*δ*_H_ 1.86 dd, *J* = 14.2, 7.0 Hz) with C-5 (*δ*_C_ 73.7, s), and H-15 (*δ*_H_ 5.83, br s) with C-17 (*δ*_C_ 63.4, t) confirmed the attachment of the hydroxyl groups to C-5 and C-17. The linkage of the hydroxyl groups to C-5 and C-17 was confirmed by the HMBC correlations from a hydroxyl group (*δ*_H_ 4.97, br s) to C-4, C-5, and C-6, from H-18 to C-5, from H-17 to C-12 and C-16, and from H-15 to C-17. The relative configuration of **1** was established based on the ROESY spectrum, which was the same as that of naviculine A (Fig. 3). Thus, the structure of **1** was elucidated with its assigned NMR spectroscopic data listed in Table 1.

**Table 1:** ^1^H and ^13^C NMR data (*δ* in ppm, *J* in Hz) of compounds **1** and **2**

No	**1**		**2**	
	*δ*_C_^a^	*δ*_H_^b^, mult (*J*)	*δ*_C_^a^	*δ*_H_^b^, mult (*J)*
1	30.0, CH_2_	2.05 (m) 1.60 (overlap)	29.6, CH_2_	1.87 (overlap) 1.59 (m)
2	21.0, CH_2_	1.67 (overlap) 1.60 (overlap)	21.1, CH_2_	1.63 (m)
3	36.8, CH_2_	2.14 (m) 1.04 (d, 12.2)	36.8, CH_2_	2.17 (overlap) 1.06 (d, 12.2)
4	38.7, C		38.7, C	
5 5-OH	73.7, C	4.97 (br s)	73.6, C	
6	31.6, CH_2_	2.87 (dt, 14.2, 7.0) 1.86 (dd, 14.2, 7.0)	31.4, CH_2_	2.87 (dt, 13.5, 7.5) 1.90 (overlap)
7	33.1, CH_2_	2.32 (td, 14.2, 7.0) 1.94 (dd, 14.2, 7.0)	31.6, CH_2_	2.66 (dd, 13.5, 7.5) 1.94 (dd, 13.5, 7.5)
8	44.4, C		45.7, C	
9	50.5, CH	1.92 (br s)	44.3, CH	2.17 (overlap)
10	47.4, C		49.0, C	
11	29.1, CH_2_	1.69 (overlap) 1.46 (m)	28.4, CH_2_	1.71 (m)
12	32.4, CH	2.55 (s)	35.5, CH	2.32 (br s)
13	42.8, CH_2_	1.66 (overlap)	37.9, CH_2_	2.44 (d, 9.2) 1.76 (m)
14	49.3, CH	2.10 (m)	46.8, CH	2.47 (br s)
15	128.6, CH	5.83 (br s)	73.9, CH	4.30 (s)
16	150.7, C		159.1, C	
17	63.4, CH_2_	4.46 (d, 2.2)	106.1, CH_2_	5.05 (overlap)
18	24.9, CH_3_	1.15 (s)	24.8, CH_3_	1.14 (s)
19	61.1, CH_2_	3.11 (br s) 2.62 (m)	61.0, CH_2_	3.10 (d, 11.0) 2.62 (d, 11.0)
20	79.4, CH	2.70 (s)	79.7, CH	2.72 (s)
1'	53.4, CH_2_	3.31 (m) 2.99 (m)	53.4, CH_2_	3.29 (dt, 12.7, 7.8) 2.98 (dt, 12.7, 7.8)
2'	33.8, CH_2_	2.80 (m)	34.0, CH_2_	2.79 (t, 6.9)
3'^**c**^	178.7, C		176.2, C	

^a^Recorded at 150 MHz, Recorded in pyridine-*d*_5_

^b^Recorded at 600 MHz, Recorded in pyridine-*d*_5_

^c^Assigned by analysis of the HMBC spectrum

Compound **2** was obtained as colorless amorphous powder. The IR spectrum showed absorptions for hydroxyl (3428 cm^−1^) and carboxyl (1603 cm^−1^) groups. Its molecular formula was assigned as C_23_H_33_NO_4_ by the analysis of its HRESIMS (*m/z* 388.2486 [M+H]^+^, calcd 388.2482), indicating eight degrees of unsaturation. Comparison of the ^1^H and ^13^C NMR spectra data of **2** with that of **1** (Table 1) revealed that **2** was an analogue of **1**. The main difference between them was that an endo double bond at C-15/C-16 and a hydroxyl group at C-17 in **1** were replaced by a double bond at C-16/C-17 and a hydroxyl group at C-15 in **2**. Compared with the ^13^C NMR data of **1**, some chemical shifts in that of **2** changed due to the shift of double bond: a high-field chemical shifts of C-7 (*δ*_C_ 31.6, t, Δ − 1.5), C-9 (*δ*_C_ 44.3, d, Δ − 6.2), C-11 (*δ*_C_ 28.4, t, Δ − 0.7), C-13 (*δ*_C_ 37.9, t, Δ − 4.9), C-14 (*δ*_C_ 46.8, d, Δ − 2.5) and a low-field chemical shifts of C-8 (*δ*_C_ 45.7, s, Δ + 1.3), C-10 (*δ*_C_ 49.0, s, Δ + 1.6), and C-12 (*δ*_C_ 35.5, d, Δ + 3.1). The main difference between **1** and **2** was further confirmed by the HMBC correlations from H-17 to C-12, C-16, and C-15, and from H-15 to C-7, C-12, and C-14. The presence of C-3' carboxyl group could be further confirmed by the HMBC correlation from both H_2_-1' and H_2_-2' to C-3'. The fragment C-1'/C-2'/C-3' connection with N-atom was confirmed by the HMBC correlation from H-20 to C-1'. The ROESY correlation of H-9*β*/Η-15 indicated that the hydroxyl group at C-15 was *α*-oriented (Fig. 3). Combined with all the evidence, the structure of compound **2** was established.

Compound **3** was isolated as colorless amorphous powder. Its molecular formula was deduced as C_32_H_42_N_2_O_4_ by the analysis of the positive HRESIMS ion peak at *m*/*z* 519.3221 ([M+H]^+^, calcd 519.3217). Its IR spectrum showed the presence of hydroxyl (3402 cm^−1^), phenyl (1583 cm^−1^, 1513 cm^−1^, 1456 cm^−1^), and carboxyl (1637 cm^−1^) groups. The ^1^H NMR data of **3** (Table 2) exhibited the signals ascribed to an imine unit (*δ*_H_ 7.60, overlap), a *p*-substituted phenyl (*δ*_H_ 7.09, d, *J* = 8.4 Hz; 7.19, overlap), a trisubstituted vinyl group (*δ*_H_ 5.79, s), an oxygen-bearing methylene (*δ*_H_ 4.62, s), a nitrogen-bearing methine (*δ*_H_ 3.75, s), two methylenes connecting nitrogen (*δ*_H_ 2.95, m; 2.67, m), and two methyls (*δ*_H_ 2.29, s; 1.16, s). The ^13^C NMR spectrum displayed the signals for two methyls, twelve methylenes (one oxygenated), ten methines (six olefinic), seven quaternary carbons (three olefinic and one oxygenated). By detailed analyses of its 2D spectra, it revealed the key ^1^H-^1^H COSY correlations of H_2_-1/H_2_-2/H_2_-3, H_2_-6/H_2_-7, H-9/H_2_-11/H-12/H_2_-13/H-14/H-20 (Fig. 2), and the key HMBC correlations of H_2_-1/C-10, H_2_-6/C-5, H_2_-7/C-8, C-9, C-14 and C-15, H-15/C-8, C-9 and C-12, H_2_-17/C-12, C-15 and C-16, H_3_-18/C-3, C-4, C-5 and C-19, and H-20/C-1, C-5, C-13, and C-19. All the above evidence showed that **3** was also a hetidine-type diterpene alkaloid. The key ^1^H-^1^H COSY correlations of H-2'/H-3', H-5'/H-6', H_2_-7'/H_2_-8', and H_2_-9'/H_2_-10', together with the key HMBC correlations from H_2_-7' to C-3', C-4', C-5', C-8', and correlations from H_3_-12' to C-8' and C-9' further confirmed the existence of this moiety. The linkage of the moiety with C-17 via a C-O bond was confirmed by the key HMBC correlation from H_2_-17 to C-1'. The carboxyl group was deduced to be linked with C-10', which accounted for the residual one degree of unsaturation and an IR absorption (1637 cm^−1^). Thus, the structure of **3** was established, and named as tangutidine C.

**Table 2:** ^1^H and ^13^C NMR data (*δ* in ppm, *J* in Hz) of compound **3**

**3**					
No	*δ*_C_^a^	*δ*_H_^b^, mult (*J*)	No	*δ*_C_^a^	*δ*_H_^b^, mult (*J*)
1	29.2, CH_2_	2.11 (m); 1.56 (overlap)	17	69.1, CH_2_	4.62 (s)
2	22.0, CH_2_	1.56 (overlap); 1.39 (m)	18	20.0, CH_3_	1.16 (s)
3	31.6, CH_2_	2.06 (overlap); 1.26 (m)	19	169.8, CH	7.60 (overlap)
4	46.0, C		20	81.3, CH	3.75 (s)
5	72.3, C		1'	158.4, C	
6	32.6, CH_2_	1.89 (m); 1.70 (overlap)	2'	115.6, CH	7.09 (d, 8.4)
7	32.2, CH_2_	2.24 (td, 13.7, 6.4) 1.80 (dd, 13.7, 6.4)	3'	130.6, CH	7.19 (overlap)
8	44.7, C		4'	133.5, C	
9	47.9, CH	2.06 (overlap)	5'	130.6, CH	7.19 (overlap)
10	46.4, C		6'	115.6, CH	7.09 (d, 8.4)
11	28.5, CH_2_	1.70 (overlap) 1.44 (m)	7'	33.5, CH_2_	2.79 (m)
12	32.7, CH	2.59 (br s)	8'	60.3, CH_2_	2.67 (m)
13	44.1, CH_2_	1.96 (dd, 11.9, 4.2) 1.64 (m)	9'	54.3, CH_2_	2.95 (m)
14	45.5, CH	1.88(m)	10'	34.5, CH_2_	2.72 (m)
15	131.7, CH	5.79 (s)	11'^**c**^		
16	146.9, C		12'	42.2, CH_3_	2.29 (s)

^a^Recorded at 200 MHz, Recorded in pyridine-*d*_5_

^b^Recorded at 800 MHz, Recorded in pyridine-*d*_5_

^c^No observed at 200 MHz in pyridine-*d*_5_

The known alkaloids (**4**–**7**) were identified to be tanaconitine [10], heteratisine [14], trolline [17] and isocorydine [18], by comparison with published physical and spectroscopic data. Alkaloids **6** and **7** were first reported from *A. tanguticum*.

Additionally, compounds **1**–**7** were evaluated for their cytotoxicity against five human cancer cell lines (HL-60, A549, SMMC-7721, MCF-7, SW480), with *cis*-platin and paclitaxel as positive controls. As a result, no compounds showed activity against the tested cell lines (Table S1).

## Experimental Section

### General

Optical rotations were measured with a JASCO P-1020 polarimeter. UV spectra were obtained using a Shimadzu UV-2401 PC spectrophotometer. A Tensor 27 spectrophotometer was used for scanning IR spectroscopy with KBr pellets. HRESIMS data was acquired on an Agilent 6540 QSTAR TOF time-of-flight mass spectrometer. 1D and 2D NMR spectra were recorded on Bruker DRX-600 spectrometers with TMS as internal standard. Chemical shifts (*δ*) are expressed in parts per million (ppm) with reference to the solvent signals. Semipreparative HPLC was performed on an Agilent 1260 liquid chromatograph with a COSMOSIL 5C18-MS-II (4.6ID × 250 mm) column. Column chromatography (CC) was performed with silica gel (80–100 and 100–200 mesh; Qingdao Marine Chemical, Inc., Qingdao, People’s Republic of China), Sephadex LH-20 (Pharmacia, Uppsala, Sweden) and SEPAFlash column (Spherical C-18, 20–45 μm, 100 *Å*m). Fractions were monitored by TLC (thin-layer chromatography) and spots were detected with the modified Dragendorff’s reagent.

### Plant Material

The dried whole plants of *Aconitum tanguticum* (Maxim.) Stapf (Ranunculaceae) were provided by Qinghai Jinke Tibetan Medicine Pharmaceutical Co. Ltd. in 2019 and identified by Prof. Yu-Bi Zhou. A voucher specimen (No. 2019-WZ-01) is deposited in Qinghai Provincial Key Laboratory of Tibetan Medicine Pharmacology and Safety Evaluation, Xining, China.

### Cytotoxicity Assay

Five human cancer cell lines, human myeloid leukemia HL-60, lung cancer A-549 cells, hepatocellular carcinoma SMMC-7721, breast cancer MCF-7, and colon cancer SW480, were purchased from the Shanghai Institute of Biochemistry and Cell Biology, Chinese Academy of Sciences (Shanghai, China). Cells were cultured according to the manufacturer’ recommendations. All mediums were supplemented with 10% fetal bovine serum (FBS), 100 units/ml penicillin G sodium and 100 μg/ml streptomycin (HyClone). All the cells were incubated at 37 °C, 5% CO_2_ in a humidified atmosphere. Cytotoxicity of compounds was determined by MTS method. Briefly, 5 × 10^3^ cells were plated in 96-well plates 12 h before treatment and continuously exposed to test compounds for 48 h. Then MTS (Promega) was added to each well. The samples were incubated at 37 °C for 1–4 h and the optical density (OD) was measured at 492 nm using a microplate reader (MULTISKAN FC). The IC_50_ values were calculated by Reed and Muench’s method [19].

### Extraction and Isolation

Dried whole plants of *A. tanguticum*. (8.8 kg) were powered and extracted with 70% EtOH (40 L each) for three times, each time for 3 days. The extract was filtered and concentrated under reduced pressure to give the crude extract. The extract was suspended in water, solution was acidified with 5% aq. HCl to pH 2.0 and the acidic solution was successively extracted with petroleum ether (PE), CHCl_3_ and *n*-BuOH. Then the acidic solution was was basified with saturated NaOH solution and extracted with CHCl_3_. The *n*-BuOH part was basified with saturated NaOH solution and extracted with CHCl_3_, EtOAc and *n*-BuOH. The EtOAc part was concentrated to yield the total crude alkaloids (28 g). The part (28 g) was applied to ODS chromatography by eluting with MeOH–H_2_O (23:67 to 100:0) to give four fractions E1–E4. Fr. E4 (12 g) was subjected to silica gel CC with CHCl_3_-Acetone-DEA (15:1:0.1 to 0:1) to afford eight fractions F1–F8. Then Fr. F3 (800 mg) was chromatographed on flash column by eluting with MeOH-H_2_O (3:7 to 10:0) to yield five fractions F3A–F3E. Fr. F3B (100 mg) was subjected on Sephadex LH-20 (CHCl_3_-MeOH, 1:1) to give subfractions B1–B6. Fr. B2 (50 mg) was purified by semi-preparative HPLC (3 ml/min, MeCN/H_2_O 15.9:84.1) to yield **1** (2.8 mg, t_R_ = 7.2 min), **2** (4.0 mg, t_R_ = 11.5 min) and **3** (1.0 mg, t_R_ = 21.1 min). Fr. F2 (1 g) was chromatographed on flash column with a MeOH-H_2_O (3:7 to 10:0) gradient system to yield five fractions F2A–F2E. Fr. F2D (18.7 mg) was purified by semi-preparative HPLC (3 ml/min, MeCN/H_2_O 20:80) to yield **6** (1.5 mg, t_R_ = 25.2 min). Fr. F2A (30 mg) was purified by semi-preparative HPLC (3 ml/min, MeCN/H_2_O 16:84) to yield **7** (20.3 mg, t_R_ = 4 min). The CHCl_3_ extract (1 g) was subjected to silica gel CC with a Acetone-PE-DEA (diethylamine) gredient system (10:1:0.1, 8:1:0.1, 5:1:0.1, 3:1:0.1, 1:1:0.1 and 1:0:0.1) to yield six frictions A–F. Fr. C (120 mg) was subjected on Sephadex LH-20 (CHCl_3_-MeOH, 1:1) to give subfrictions C1–C5. Fr. C3 (25 mg) was purified by semi-preparative HPLC (3 ml/min, MeCN/H_2_O/1‰ triethylamine 63:37) to yield **4** (19.5 mg, t_R_ = 15.6 min). Fr. F (56.7 mg) was purified by semi-preparative HPLC (3 ml/min, MeCN/H_2_O/1 ‰ triethylamine 55:45) to yield **5** (15.3 mg, t_R_ = 45 min).

### Physical Constants and Spectroscopic Data of Compounds 1–3

#### Tangutidine A (**1**)

Colorless oil; ${[\alpha ]}_{\mathrm{D}}^{21}$ − 21.2 (*c* 0.10, MeOH); UV (MeOH) λ_max_ (log ε) 196 (4.90) nm; For ^1^H NMR and ^13^C NMR spectroscopic data, see Table 1; HRESIMS *m/z* 388.2483 [M+H]^+^ (calcd for C_23_H_34_NO_4_, 388.2482).

#### Tangutidine B (**2**)

Colorless amorphous powder; ${[\alpha ]}_{\mathrm{D}}^{21}$ − 50.8 (*c* 0.09, MeOH); UV (MeOH) λ_max_ (log ε) 196 (5.02) nm; For ^1^H NMR and ^13^C NMR spectroscopic data, see Table 1; HRESIMS *m/z* 388.2486 [M+H]^+^ (calcd for C_23_H_34_NO_4_, 388.2482).

#### Tangutidine C (**3**)

Colorless amorphous powder; ${[\alpha ]}_{\mathrm{D}}^{22}$ 19.4 (*c* 0.10, MeOH); UV (MeOH) λ_max_ (log ε) 196 (5.36), 248 (3.85), 275 (4.05) nm; For ^1^H NMR and ^13^C NMR spectroscopic data, see Table 2; HRESIMS *m/z* 519.3221 [M+H]^+^ (calcd for C_32_H_43_N_2_O_4_, 519.3217).

## Supplementary Information

Below is the link to the electronic supplementary material.

Supplementary file1 (DOCX 14120 kb)
