# Supplementary material for: Tangutidines A–C, Three Amphoteric Diterpene Alkaloids from Aconitum tanguticum
Source: Nat Prod Bioprospect. 2021 May 12;11(4):459–64. doi: 10.1007/s13659-021-00310-3 (PMC8275809; doi:10.1007/s13659-021-00310-3)
Supplement: Supplementary file 1 — Supplementary file1 (DOCX 14120 kb) [file 13659_2021_310_MOESM1_ESM.docx]

**Supplementary Material**

**Tangutidines A–C, three amphoteric diterpenoid alkaloids from *Aconitum tanguticum***

Hao-Yi Li^1,2^ · Bing-Chao Yan^1^ · Li-Xin Wei^3^ ·Han-Dong Sun^1*^· Pema-Tenzin Puno^1*^

*^1^ State Key Laboratory of Phytochemistry and Plant Resources in West China, Kunming Institute of Botany, Chinese Academy of Sciences, and Yunnan Key Laboratory of Natural Medicinal Chemistry, Kunming 650201, People’s Republic of China*

*^2^ University of Chinese Academy of Sciences, Beijing 100049, People’s Republic of China*

*^3^ Q**inghai Provincial Key Laboratory of Tibetan Medicine Pharmacology and Safety Evaluation,* *Northwest Institute of Plateau Biology, Chinese Academy of Sciences, Xining 810008, People’s Republic of China*

**Contents of Supplementary Material**

[Figure S1. ^1^H NMR spectrum of compound 1 (pyridine-*d*_5_, 600 MHz) 2](#_Toc66485277)

[Figure S2. ^13^C NMR spectrum of compound 1 (pyridine-*d_5_*, 151 MHz) 3](#_Toc66485278)

[Figure S3. HSQC spectrum of compound 1 4](#_Toc66485279)

[Figure S4. ^1^H-^1^H COSY spectrum of compound 1 4](#_Toc66485280)

[Figure S5. HMBC spectrum of compound 1 5](#_Toc66485281)

[Figure S6. ROESY spectrum of compound 1 7](#_Toc66485282)

[Figure S7. HRESIMS spectrum of compound 1 8](#_Toc66485283)

[Figure S8. ORD spectrum of compound 1 8](#_Toc66485284)

[Figure S9. IR spectrum of compound 1 9](#_Toc66485285)

[Figure S10. UV spectrum of compound 1 9](#_Toc66485286)

[Figure S11. CD spectrum of compound 1 10](#_Toc66485287)

[Figure S12. ^1^H NMR spectrum of compound 2 (pyridine-*d*_5_, 600 MHz) 11](#_Toc66485288)

[Figure S13. ^13^C NMR spectrum of compound 2 (pyridine-*d*_5_, 151 MHz) 12](#_Toc66485289)

[Figure S14. HSQC spectrum of compound 2 13](#_Toc66485290)

[Figure S15. ^1^H-^1^H COSY spectrum of compound 2 13](#_Toc66485291)

[Figure S16. HMBC spectrum of compound 2 14](#_Toc66485292)

[Figure S17. ROESY spectrum of compound 2 17](#_Toc66485294)

[Figure S18. HRESIMS spectrum of compound 2 18](#_Toc66485295)

[Figure S19. ORD spectrum of compound 2 18](#_Toc66485296)

[Figure S20. IR spectrum of compound 2 19](#_Toc66485297)

[Figure S21. UV spectrum of compound 2 19](#_Toc66485298)

[Figure S22. CD spectrum of compound 2 20](#_Toc66485299)

[Figure S23. ^1^H NMR spectrum of compound 3 (pyridine-*d*_5_, 800 MHz) 21](#_Toc66485300)

[Figure S24. ^13^C NMR spectrum of compound 3 (pyridine-*d*_5_, 201 MHz) 22](#_Toc66485301)

[Figure S25. HSQC spectrum of compound 3 23](#_Toc66485302)

[Figure S26. ^1^H-^1^H COSY spectrum of compound 3 23](#_Toc66485303)

[Figure S27. HMBC spectrum of compound 3 24](#_Toc66485304)

[Figure S28. ROESY spectrum of compound 3 26](#_Toc66485305)

[Figure S29. HRESIMS spectrum of compound 3 27](#_Toc66485306)

[Figure S30. ORD spectrum of compound 3 27](#_Toc66485307)

[Figure S31. IR spectrum of compound 3 28](#_Toc66485308)

[Figure S32. UV spectrum of compound 3 28](#_Toc66485309)

[Figure S33. CD spectrum of compound 3 29](#_Toc66485310)

[Table S1 Cytotoxic activities of 1–3 against five human tumor cell lines at 40 *μ*M 30](#_Toc66485311)

Figure S1. ^1^H NMR spectrum of compound **1** (pyridine-*d*_5_, 600 MHz)

Figure S2 (A). ^13^C NMR spectrum of compound **1** (pyridine-*d_5_*, 151 MHz)

Figure S2 (B). ^13^C NMR spectrum of compound **1** (pyridine-*d_5_*, 151 MHz)

# Figure S3. HSQC spectrum of compound 1

# Figure S4. ^1^H-^1^H COSY spectrum of compound 1

Figure S5 (A). HMBC spectrum of compound **1**

Figure S5 (B). HMBC spectrum of compound **1**

Figure S5 (C). HMBC spectrum of compound **1**

Figure S5 (D). HMBC spectrum of compound **1**

Figure S6 (A). ROESY spectrum of compound **1**

Figure S6 (B). ROESY spectrum of compound **1**


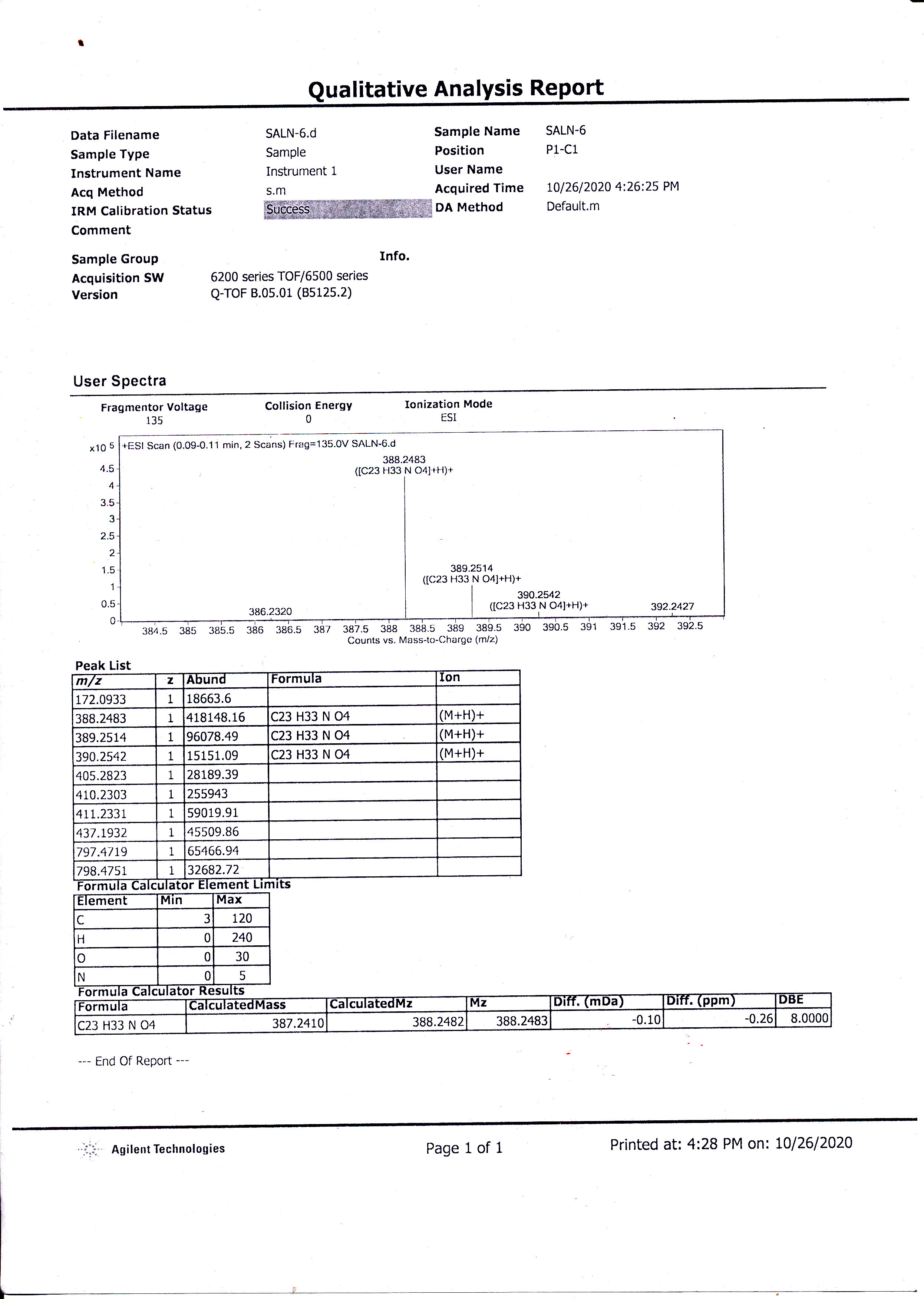


Figure S7. HRESIMS spectrum of compound **1**


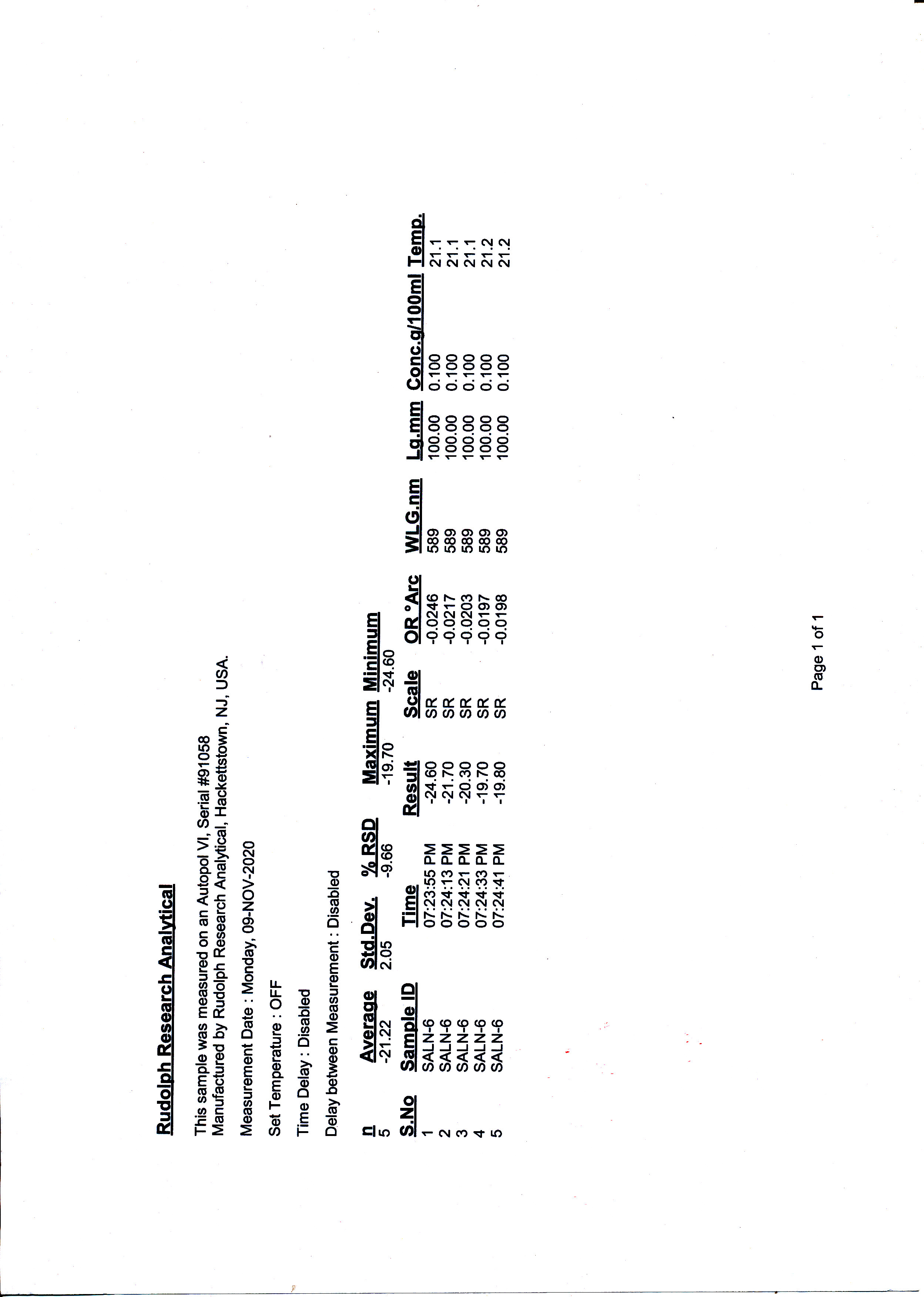


Figure S8. ORD spectrum of compound **1**


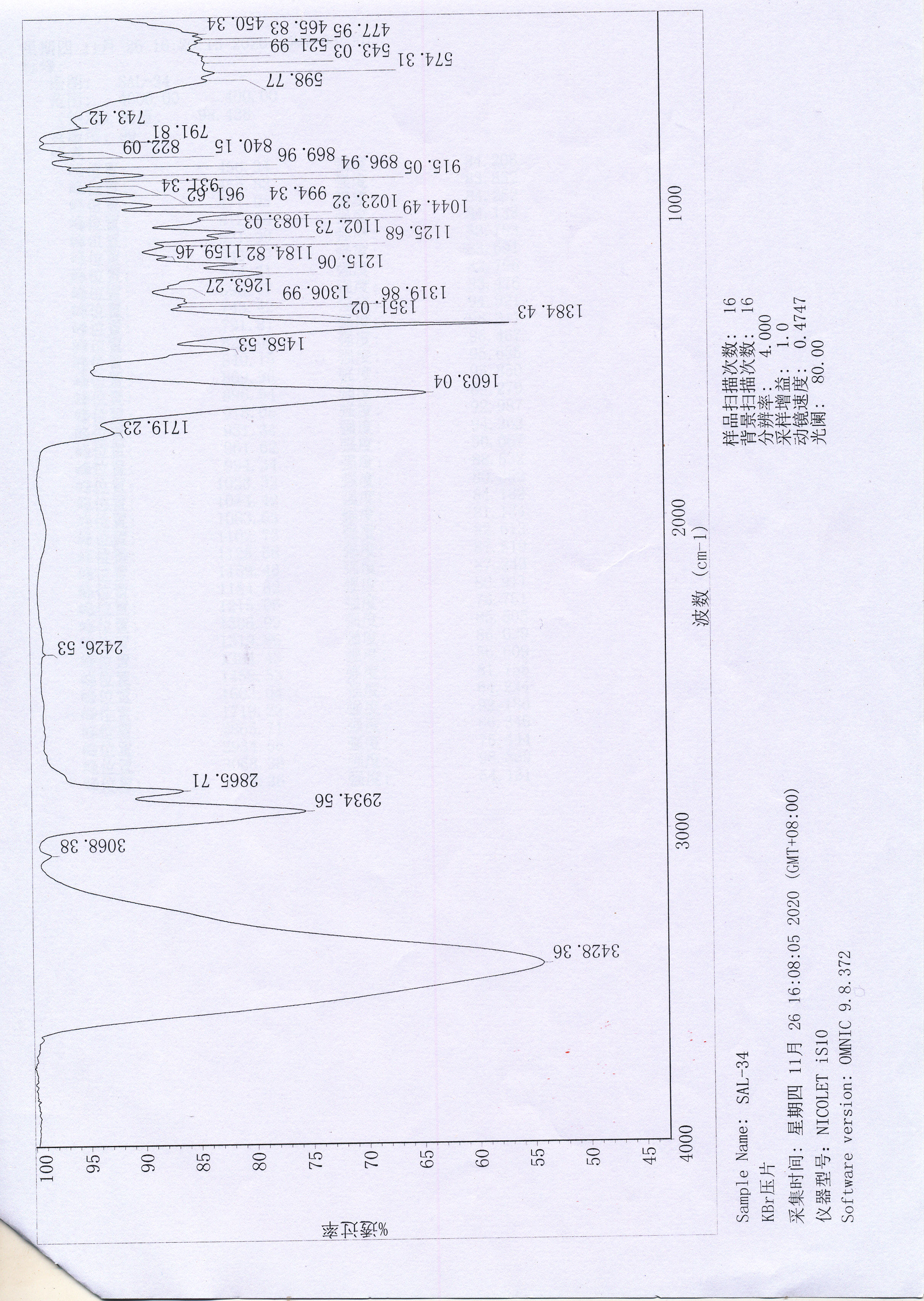


Figure S9. IR spectrum of compound **1**


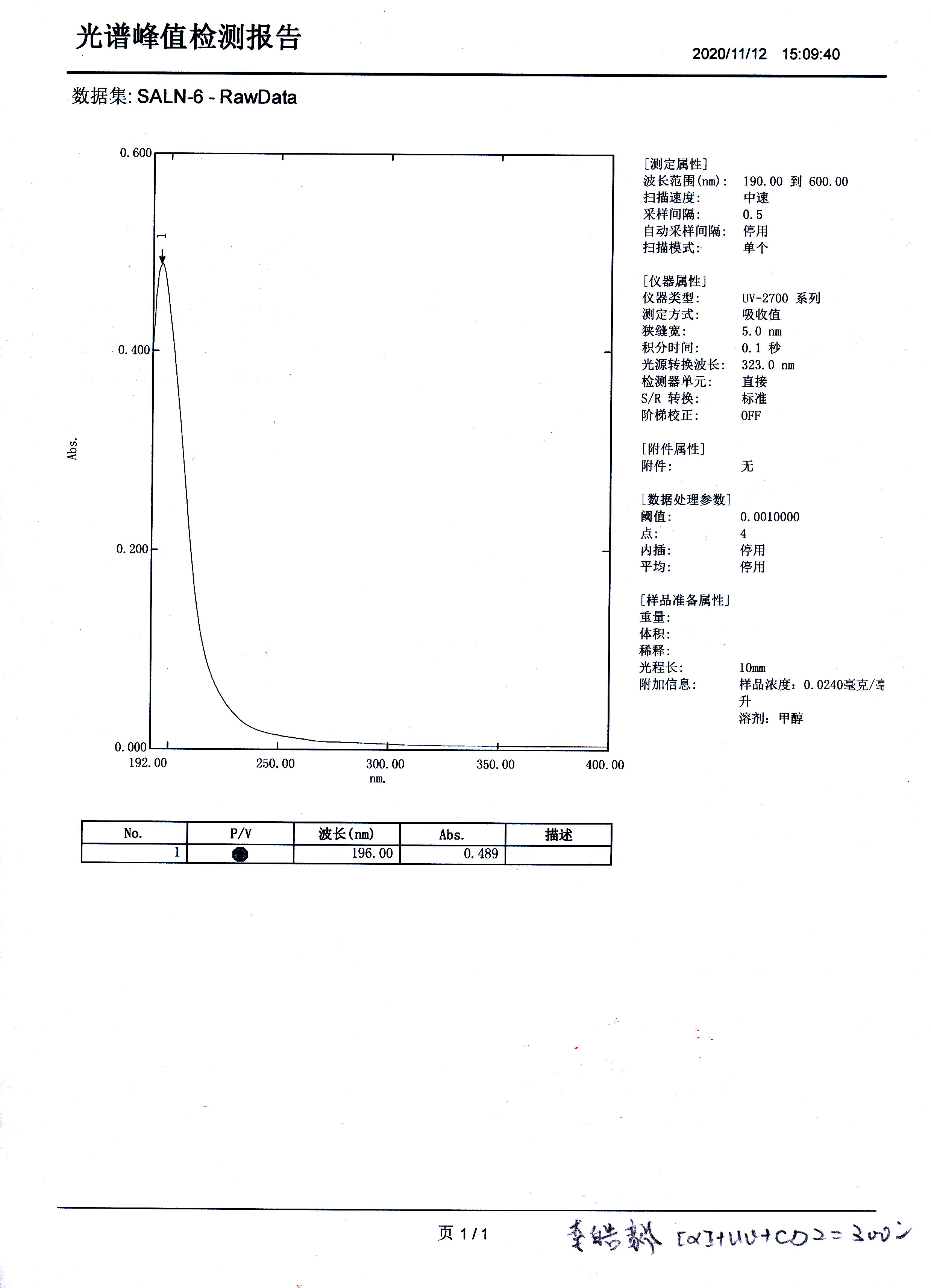


Figure S10. UV spectrum of compound **1**


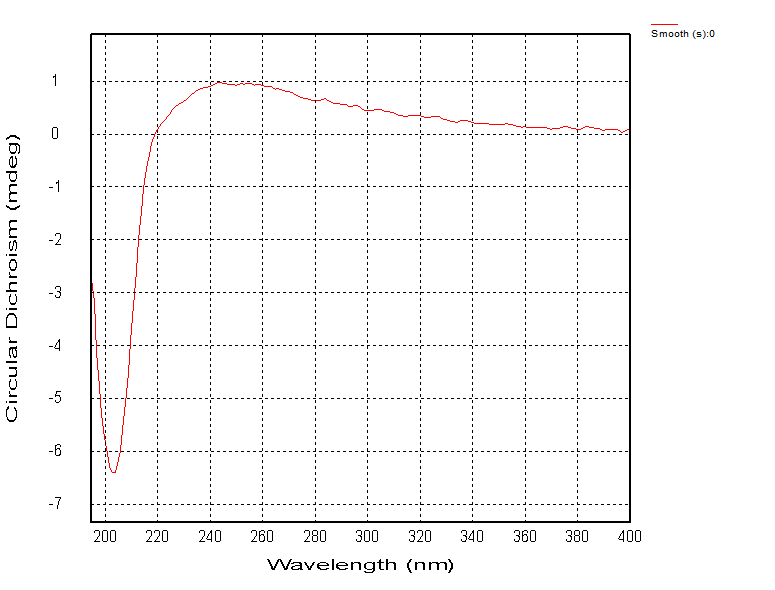


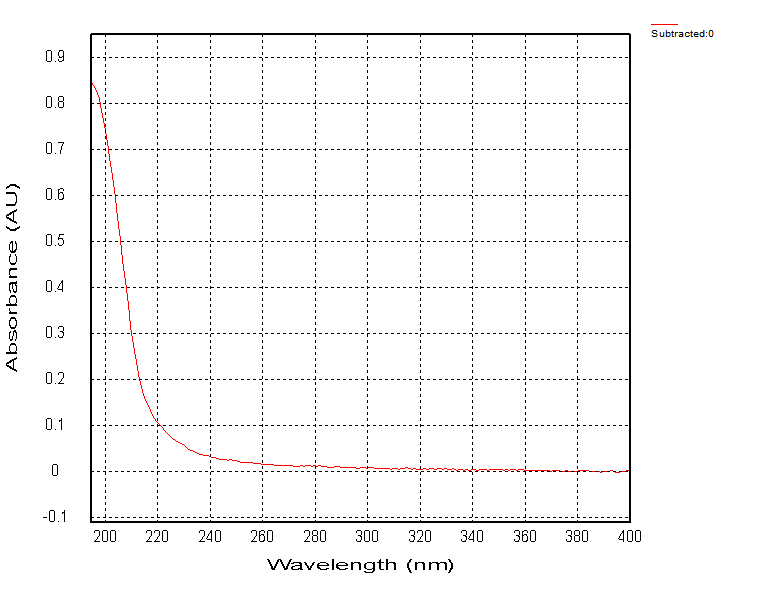


Figure S11. CD spectrum of compound **1**

Figure S12 (A). ^1^H NMR spectrum of compound **2** (pyridine-*d*_5_, 600 MHz)

Figure S12 (B). ^1^H NMR spectrum of compound **2** (pyridine-*d*_5_, 600 MHz)

Figure S13 (A). ^13^C NMR spectrum of compound **2** (pyridine-*d*_5_, 151 MHz)

Figure S13 (B). ^13^C NMR spectrum of compound **2** (pyridine-*d*_5_, 151 MHz)

Figure S14. HSQC spectrum of compound **2**

Figure S15. ^1^H-^1^H COSY spectrum of compound **2**

Figure S16 (A). HMBC spectrum of compound **2**

Figure S16 (B). HMBC spectrum of compound **2**

Figure S16 (C). HMBC spectrum of compound **2**

Figure S16 (D). HMBC spectrum of compound **2**

Figure S16 (E). HMBC spectrum of compound **2**

Figure S16 (F). HMBC spectrum of compound **2**

Figure S17 (A). ROESY spectrum of compound **2**

Figure S17 (B). ROESY spectrum of compound **2**


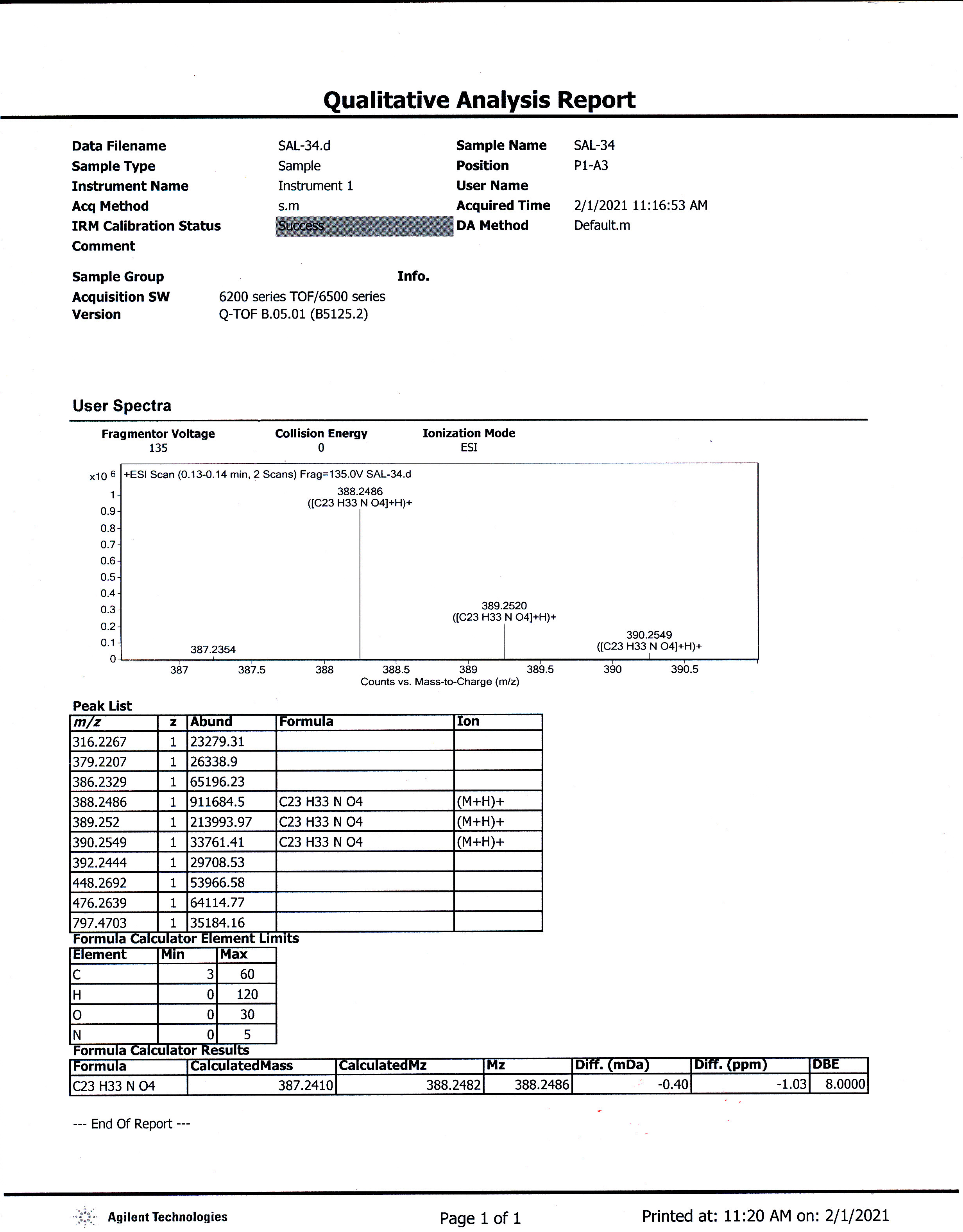


Figure S18. HRESIMS spectrum of compound **2**


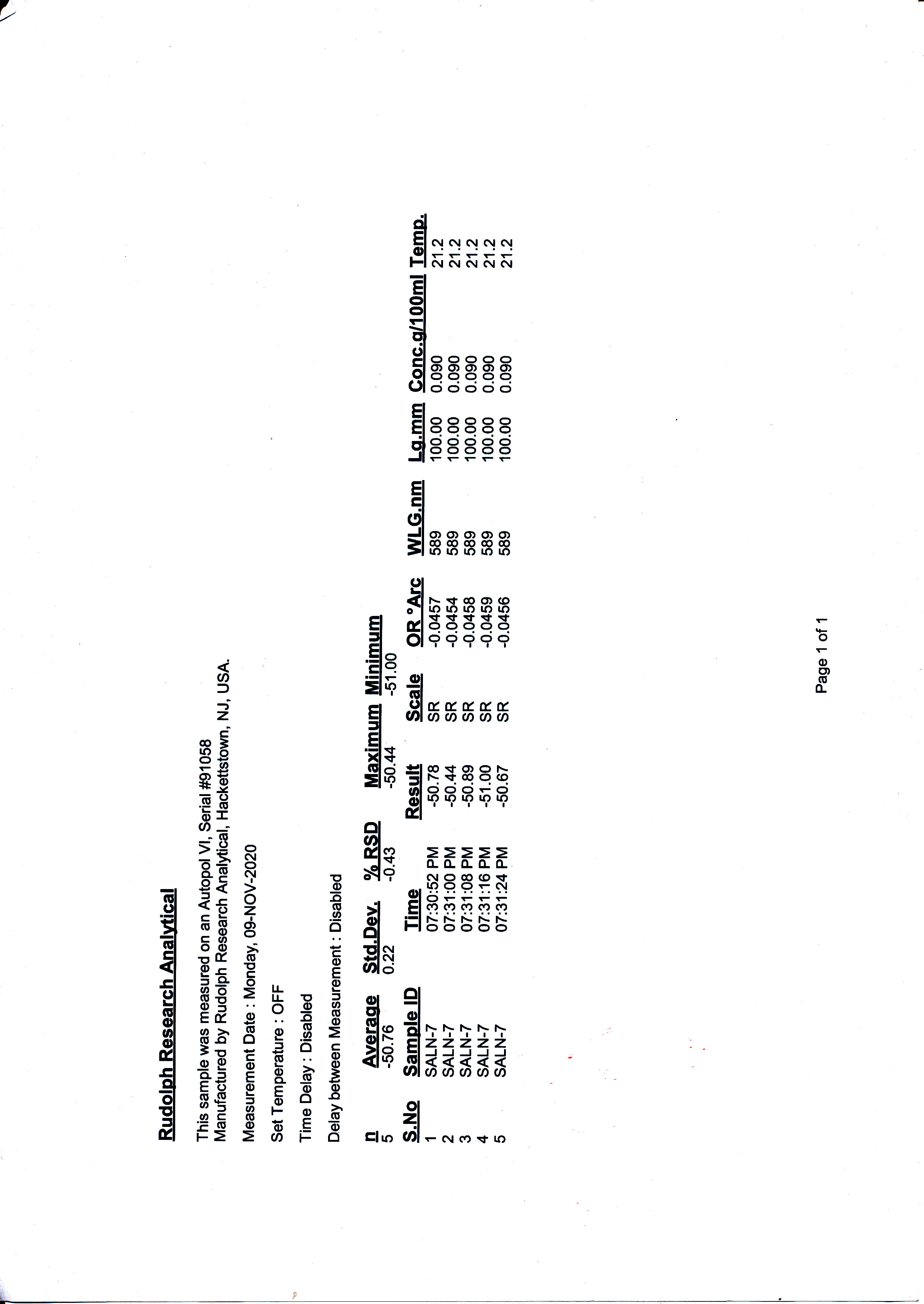


Figure S19. ORD spectrum of compound **2**


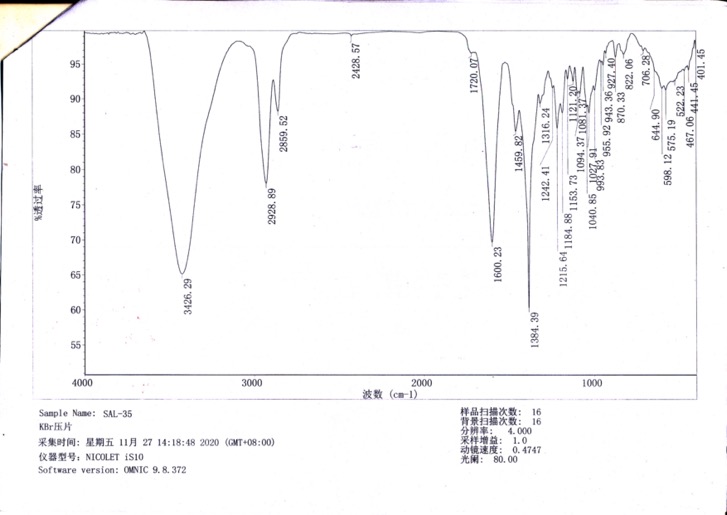


Figure S20. IR spectrum of compound **2**


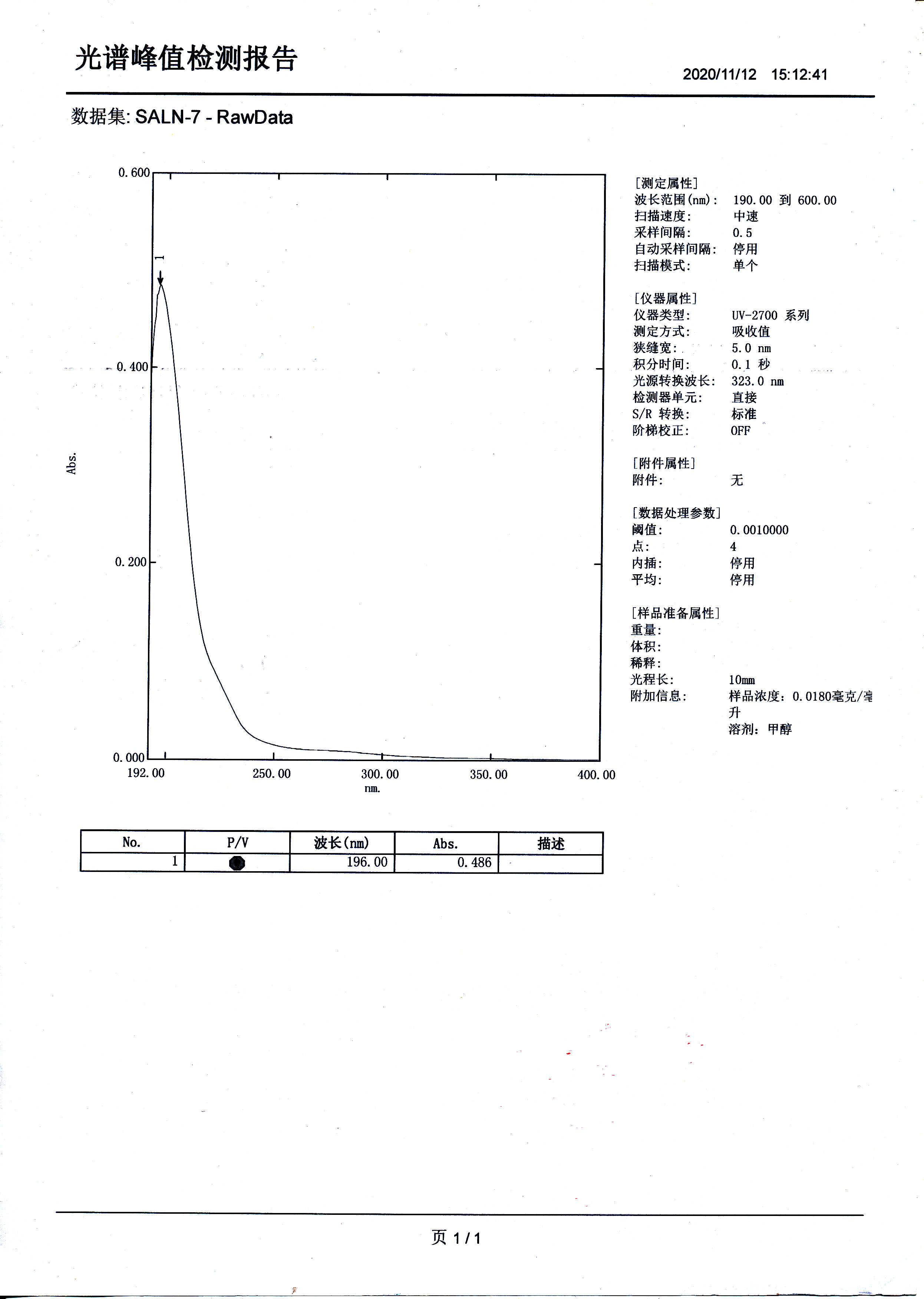


Figure S21. UV spectrum of compound **2**


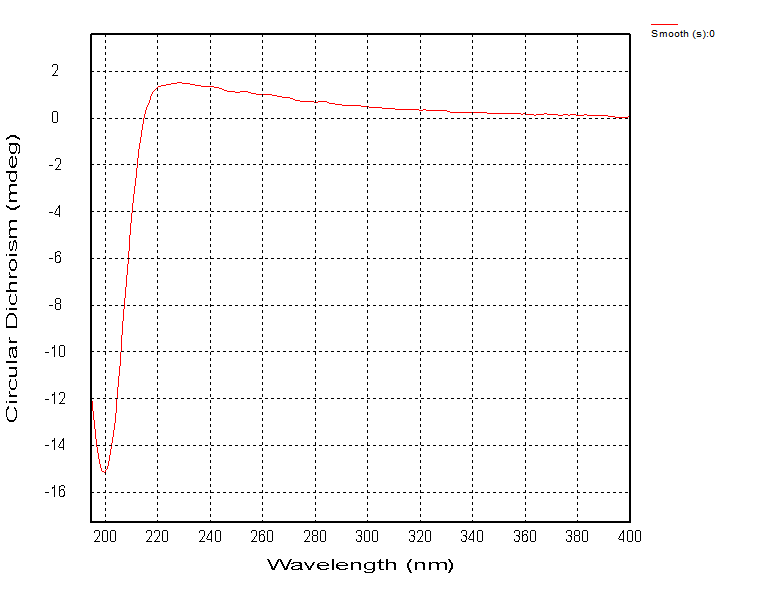


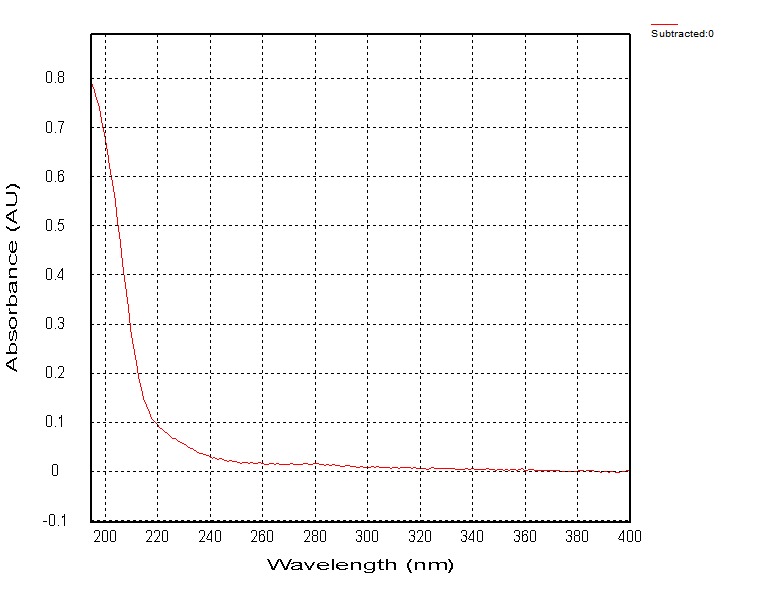


Figure S22. CD spectrum of compound **2**

Figure S23 (A). ^1^H NMR spectrum of compound **3** (pyridine-*d*_5_, 800 MHz)

Figure S23 (B). ^1^H NMR spectrum of compound **3** (pyridine-*d*_5_, 800 MHz)

Figure S24 (A). ^13^C NMR spectrum of compound **3** (pyridine-*d*_5_, 201 MHz)

Figure S24 (B). ^13^C NMR spectrum of compound **3** (pyridine-*d*_5_, 201 MHz)

Figure S25. HSQC spectrum of compound **3**

Figure S26 (A). ^1^H-^1^H COSY spectrum of compound **3**

Figure S26 (B). ^1^H-^1^H COSY spectrum of compound **3**

Figure S27 (A). HMBC spectrum of compound **3**

Figure S27 (B). HMBC spectrum of compound **3**

Figure S27 (C). HMBC spectrum of compound **3**

Figure S28 (A). ROESY spectrum of compound **3**

Figure S28 (B). ROESY spectrum of compound **3**


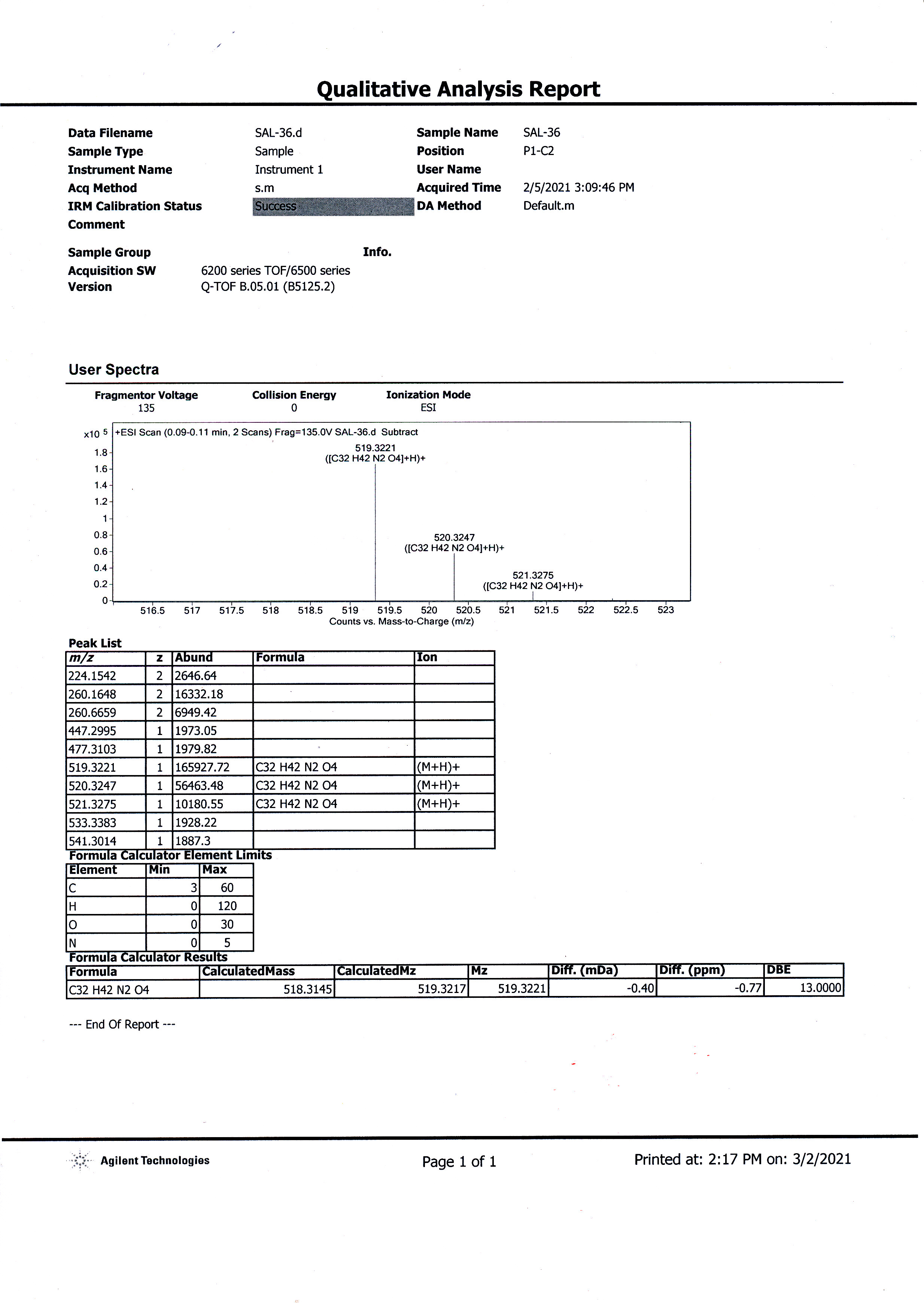


Figure S29. HRESIMS spectrum of compound **3**


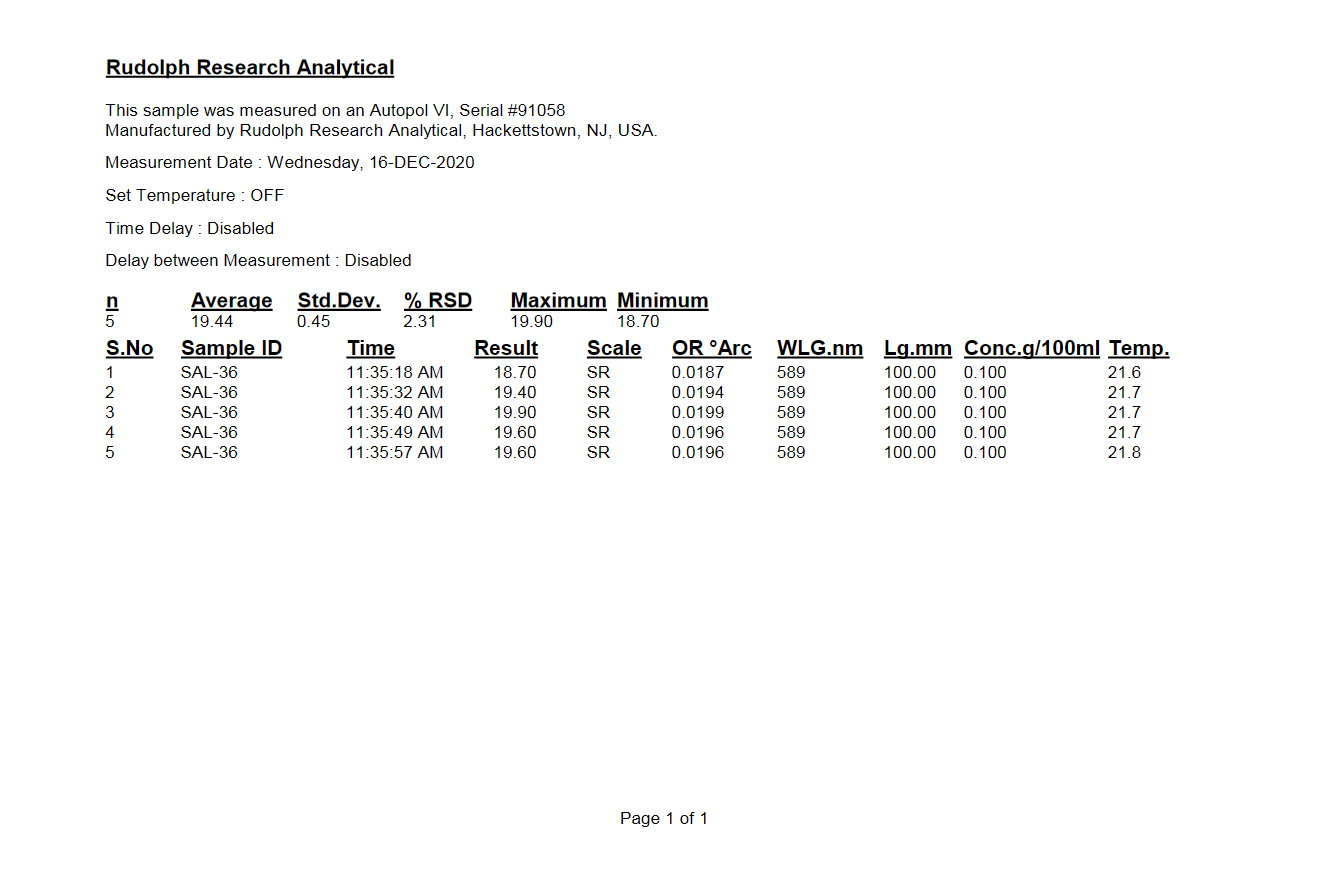


Figure S30. ORD spectrum of compound **3**


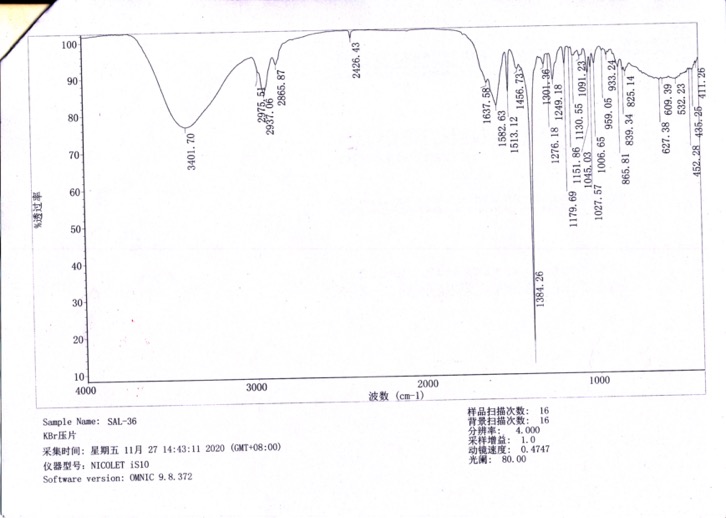


Figure S31. IR spectrum of compound **3**


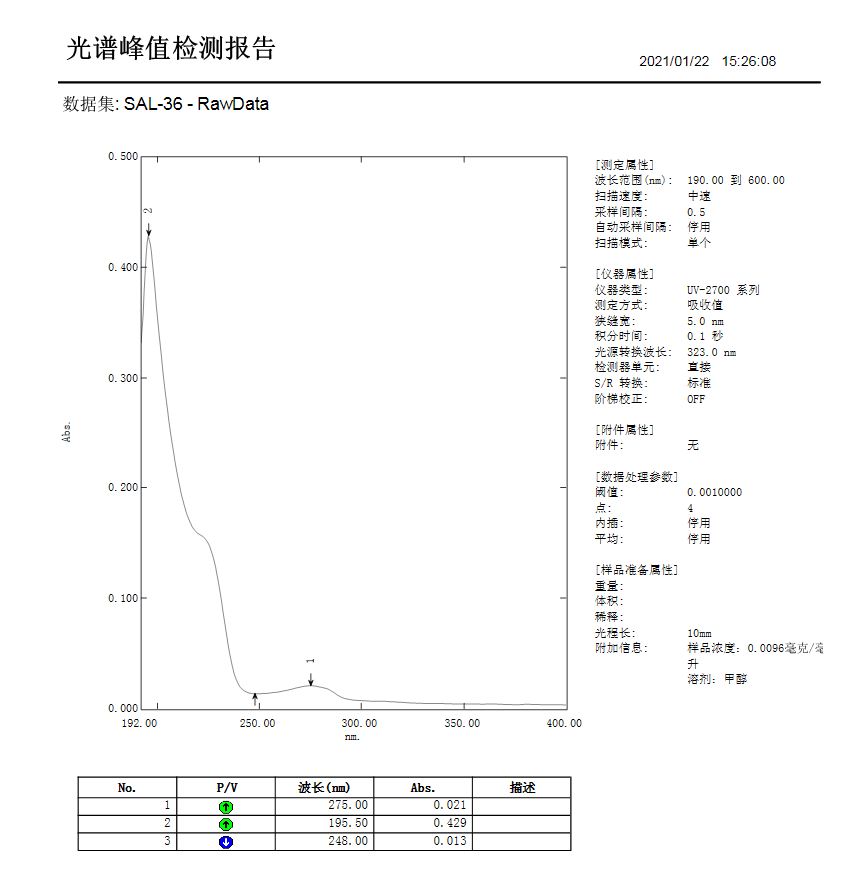


Figure S32. UV spectrum of compound **3**


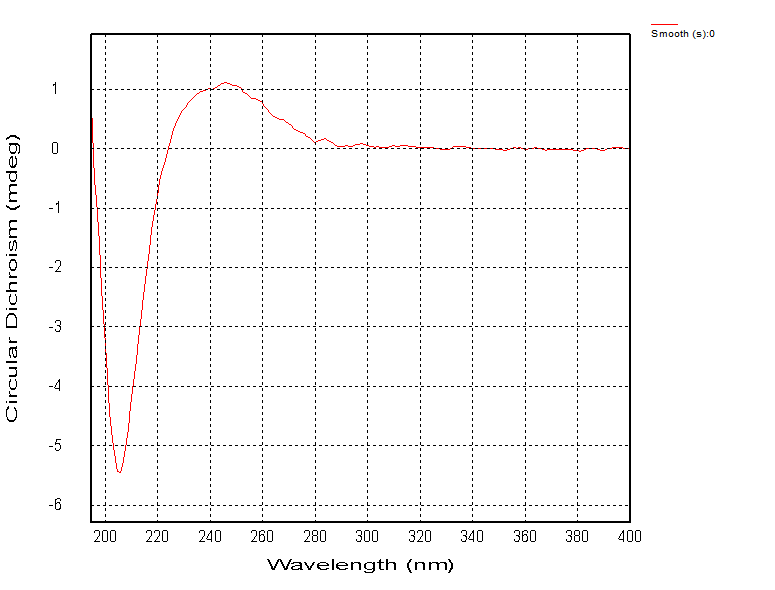


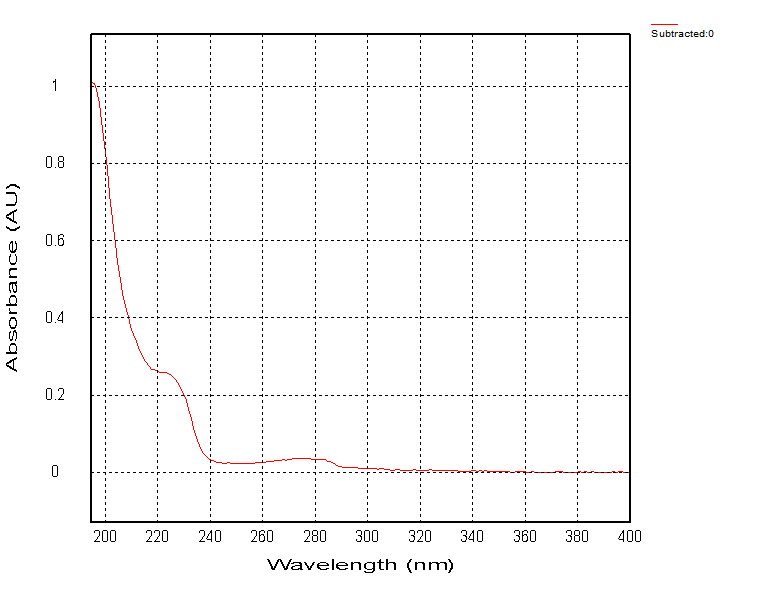


Figure S33. CD spectrum of compound **3**

Table S1 Cytotoxic activities of **1**–**3** against five human tumor cell lines at 40 *μ*M

| No. | 白血病  HL-60 | | 肺癌  A549 | | 肝癌  SMMC-7721 | | 乳腺癌  MCF-7 | | 结肠癌  SW480 | |
| --- | --- | --- | --- | --- | --- | --- | --- | --- | --- | --- |
|  | average ^a^ | SD | average ^a^ | SD | average ^a^ | SD | average ^a^ | SD | average ^a^ | SD |
| 1 | 8.70 | 2.49 | 16.73 | 0.96 | 15.79 | 2.38 | 15.34 | 3.12 | 22.85 | 3.05 |
| 2 | 12.23 | 0.96 | 21.21 | 0.63 | 16.37 | 0.46 | 30.59 | 3.88 | 28.89 | 3.45 |
| 3 | 11.92 | 0.98 | 23.72 | 1.45 | 13.58 | 3.34 | 30.01 | 1.97 | 13.25 | 3.00 |
| 顺铂 | 86.62 |  | 95.7 |  | 92.10 |  | 63.87 |  | 85.63 |  |
| 紫杉醇 | > 99.00 |  | >73.00 |  | >75.60 |  | >61.00 |  | >60.50 |  |

^a^ Cell inhibition (%)
